# Supplementary material for: Life satisfaction and happiness in patients shielding from the COVID-19 global pandemic: A randomised controlled study of the ‘mood as information’ theory
Source: PLoS One. 2020 Dec 11;15(12):e0243278. doi: 10.1371/journal.pone.0243278 (PMC7732102; doi:10.1371/journal.pone.0243278)
Supplement: Attachment — Submitted filename: Response to reviewers.docx [file pone.0243278.s002.docx]

Response to reviewers:

Dear Reviewers,

Thank you for your correspondence and for considering our study for publication. We are delighted that you agree our study holds merit and have provided us with such helpful comments. As a group we have met and discussed your comments in detail and worked through them in depth, hopefully addressing each point. We have revised the manuscript accordingly and would like to resubmit the revised version for your consideration. Below we have written our response to each of the comments provided.

Thank you again for your helpful comments and we look forward to hearing from you soon.

Journal requirements

Set 1:

**1. Please ensure that your manuscript meets PLOS ONE's style requirements, including those for file naming. The PLOS ONE style templates can be found at**

**https://journals.plos.org/plosone/s/file?id=wjVg/PLOSOne_formatting_sample_main_body.pdf and**

**https://journals.plos.org/plosone/s/file?id=ba62/PLOSOne_formatting_sample_title_authors_affiliations.pdf**

Thank you for clarifying the style requirements PLOS ONE requires. In response to this comment we have altered the formatting to ensure that it complies with your requirements. We hope this satisfies your request.

**2. Thank you for stating the following in the Funding Section of your manuscript:**

**"This work was funded by the renal department at the Queen Elizabeth
Hospital. This research was independent from external funders." We note that you have provided funding information that is not currently declared in your Funding Statement. However, funding information should not appear in the Acknowledgments section or other areas of your manuscript. We will only publish funding information present in the Funding Statement section of the online submission form.**

**Please remove any funding-related text from the manuscript and let us know how you would like to update your Funding Statement. Currently, your Funding Statement reads as follows: "No. The funders had no role in study design, data collection and analysis, decision to publish, or preparation of the manuscript."**

**Please include your amended statements within your cover letter; we will change the online submission form on your behalf.**

Thank you for this point. In reply to this, we have removed the funding statements from within the text. Please update our funding statement to the following: ‘This work was funded by the renal department at the Queen Elizabeth Hospital. This research was independent from external funders’. In addition, our cover letter now says: ‘This work was funded by the renal department at the Queen Elizabeth Hospital. This research was independent from external funders’.

We hope this is satisfactory and meets your request.

**3. We note that you have indicated that data from this study are available upon request. PLOS only allows data to be available upon request if there are legal or ethical restrictions on sharing data publicly. For information on unacceptable data access restrictions, please see http://journals.plos.org/plosone/s/data-availability#loc-unacceptable-data-access-restrictions.**

**In your revised cover letter, please address the following prompts:**

**a) If there are ethical or legal restrictions on sharing a de-identified data set, please explain them in detail (e.g., data contain potentially identifying or sensitive patient information) and who has imposed them (e.g., an ethics committee). Please also provide contact information for a data access committee, ethics committee, or other institutional body to which data requests may be sent.**

Thank you very much for this comment. We have made this data as a supplementary information file.

**b) If there are no restrictions, please upload the minimal anonymized data set necessary to replicate your study findings as either Supporting Information files or to a stable, public repository and provide us with the relevant URLs, DOIs, or accession numbers. Please see http://www.bmj.com/content/340/bmj.c181.long for guidelines on how to de-identify and prepare clinical data for publication. For a list of acceptable repositories, please see http://journals.plos.org/plosone/s/data-availability#loc-recommended-repositories.**

**We will update your Data Availability statement on your behalf to reflect the information you provide.**

**4. We note that you have included the phrase “data not shown” in your manuscript. Unfortunately, this does not meet our data sharing requirements. PLOS does not permit references to inaccessible data. We require that authors provide all relevant data within the paper, Supporting Information files, or in an acceptable, public repository. Please add a citation to support this phrase or upload the data that corresponds with these findings to a stable repository (such as Figshare or Dryad) and provide and URLs, DOIs, or accession numbers that may be used to access these data. Or, if the data are not a core part of the research being presented in your study, we ask that you remove the phrase that refers to these data.**

Thank you for pointing this out and we fully appreciate this comment. In response to this, we believe it simpler to simply state the lack of difference in these parameters (as one would expect in a randomised study) rather than subsume the reader in unnecessary data, either in the article itself or supplementary. This was agreed between all authors to be the best approach, and we hope this meets with your approval. We have removed the phrase “data not shown” that relates to this data in response to your comment.

**5. Your ethics statement should only appear in the Methods section of your manuscript. If your ethics statement is written in any section besides the Methods, please delete it from any other section.**

We thank you for clarifying this for us. We have now only included an ethical statement in the methods section of our paper.

Set 2

**1. Results are repeated three times: within the text, in a table form and in a figure. This is unnecessary. I personally would prefer showing figure 1. The description within the text can then be shortened as follows:**

**A summary of the results of the 4 questions in the primed and no-primed groups can be seen in Figure 1. The answers to all questions across the groups were normally distributed. Differences observed between the two groups in overall happiness (t198=3.77; p=0.0002), desire to change (t198=3.78; p=0.0002), satisfaction with life as a whole (t198=3.93; p=0.0001) and happiness in this moment (t198=2.08; p=0.04) were all significant.**

Thank you very much for this comment. We have replaced the summary of the results to comply with the above recommendation. Thank you again for this suggestion.

**2. In figure 1, the authors provide the standard error. The authors should have drawn the standard deviation instead. Using the stars system with explanation provided just below the graph, the readers can understand which differences between columns were significant: * <0.05; ** <0.01; *** <0.001.**

Thank you very much for this comment. Figure 1 has been changed to reflect the standard deviation rather than the standard error. This figure has replaced the previous figure and we hope you are happy with this change.

**3. While revising your submission, please upload your figure files to the Preflight Analysis and Conversion Engine (PACE) digital diagnostic tool,** [**https://pacev2.apexcovantage.com/**](https://pacev2.apexcovantage.com/)**. PACE helps ensure that figures meet PLOS requirements. To use PACE, you must first register as a user. Registration is free. Then, login and navigate to the UPLOAD tab, where you will find detailed instructions on how to use the tool. If you encounter any issues or have any questions when using PACE, please email PLOS at** [**figures@plos.org**](mailto:figures@plos.org)**. Please note that Supporting Information files do not need this step.**

Thank you for notifying us about this tool, we have now uploaded the files to PACE.
